# Targeting the dorsolateral prefrontal cortex to enhance memory control: divergent effects on social and non-social memories

**DOI:** 10.1093/scan/nsaf052

**Published:** 2025-05-15

**Authors:** Hui Xie, Jialin Liang, Yun Luo, Weimao Chen, Xiaoqing Hu, Dandan Zhang

**Affiliations:** Institute for Brain Research and Rehabilitation, South China Normal University, Guangzhou 510631, China; Department of Psychology, The University of Hong Kong, Hong Kong 999077, China; School of Psychology, Shenzhen University, Shenzhen 518060, China; School of Psychology, Shenzhen University, Shenzhen 518060, China; School of Psychology, Shenzhen University, Shenzhen 518060, China; Department of Psychology, The University of Hong Kong, Hong Kong 999077, China; The State Key Laboratory of Brain and Cognitive Sciences, The University of Hong Kong, Hong Kong 999077, China; Institute of Brain and Psychological Sciences, Sichuan Normal University, Chengdu 610066, China

**Keywords:** directed forgetting paradigm, emotional memory, rTMS, social memory, voluntary forgetting

## Abstract

Voluntary forgetting, governed by top-down inhibitory control in the prefrontal cortex, plays a critical role in adaptive memory regulation. This study investigated the causal role of the right dorsolateral prefrontal cortex (rDLPFC) in the forgetting of social and non-social memories. Employing high-frequency (10 Hz) repetitive transcranial magnetic stimulation (rTMS) in an offline protocol, we modulated rDLPFC activity (Active TMS condition) and compared it to a Control TMS condition targeting the vertex. Participants completed a directed forgetting (DF) task framed in social and non-social contexts. Results revealed a dissociation in rDLPFC involvement: stimulation significantly enhanced the forgetting of negative non-social memories but did not affect social memories. Furthermore, rTMS moderated the relationship between social anxiety and forgetting performance: individuals with higher social anxiety struggled to forget negative social feedback in the Control TMS condition, a difficulty alleviated by rDLPFC stimulation. These findings suggest that voluntary forgetting of social and non-social memories engages distinct neural mechanisms and highlighting rDLPFC stimulation as a potential intervention for reducing maladaptive memory biases associated with social anxiety.

## Introduction

Not all memories are equally desired. The ability to voluntarily forget unwanted memories, a phenomenon termed *voluntary forgetting* (Anderson and Hanslmayr 2014, Hu et al. 2017), serves a critical adaptive function in human cognition and emotional well-being (Nørby 2015, Fawcett et al. 2024). By suppressing distracting or distressing memories, individuals can focus on current priorities and mitigate the negative impact of past experiences on emotional health (Engen and Anderson 2018). Conversely, difficulties in memory control are linked to heightened susceptibility to psychiatric conditions such as depression, social anxiety, and post-traumatic stress disorder (Mary et al. 2020, Costanzi et al. 2021, Stramaccia et al. 2021, Seinsche et al. 2023). Investigating the mechanisms underlying voluntary forgetting and exploring strategies to enhance this capacity are therefore of profound theoretical and clinical relevance.

Voluntary forgetting is driven by top-down inhibitory processes that suppress the encoding and retrieval of undesired information (Anderson and Hanslmayr 2014, Anderson and Hulbert 2021). The prefrontal cortex, particularly the right dorsolateral prefrontal cortex (rDLPFC), plays a key role in this process, modulating activity in memory-related regions such as the hippocampus (Levy and Anderson 2012, Rizio and Dennis 2013, Oehrn et al. 2018). The item-method *directed forgetting* (DF) paradigm (Bjork 1989) is a widely used experimental framework for studying voluntary forgetting during encoding. In this task, participants are presented with items followed by cues indicating whether each item is to-be-remembered (TBR) or to-be-forgotten (TBF). Superior recall of TBR items over TBF items, known as the DF effect, reflects the efficacy of memory control mechanisms (Basden and Basden 2013).

Neuroimaging studies have consistently shown greater activation in the prefrontal cortex during attempts to forget compared to attempts to remember (Wylie et al. 2008, Nowicka et al. 2011, Yang et al. 2016, Gamboa et al. 2018), with successful forgetting linked to enhanced rDLPFC-mediated downregulation of hippocampus (Rizio and Dennis 2013, Oehrn et al. 2018). These findings demonstrate the rDLPFC’s crucial role in non-social memory control. However, the mechanisms underlying voluntary forgetting of socially significant memories remain largely unexplored.

Humans are inherently social, and many unwanted memories originate from interpersonal experiences (Nørby 2018, Rohde et al. 2018, Xie et al. 2021). While most DF research focuses on non-social content, studies suggest that people have a unique capacity to spontaneously forget negative social feedback to preserve self­esteem. This phenomenon, known as *mnemic neglect* (Sedikides et al. 2016), is often attributed to insufficient encoding of self-threatening social information (Zengel et al. 2018, Rigney et al. 2021, Yao et al. 2021). However, individuals with affective disorders, such as depression and social anxiety, struggle to forget negative self­relevant social memories, leading to persistent emotional distress (Saunders 2011, Zengel et al. 2015). Developing strategies to enhance the active forgetting of social memories could have significant implications for reducing cognitive and emotional burdens associated with such conditions (Einarsen and Mikkelsen 2002, Fung and Alden 2017, Rappaport and Barch 2020).

Non-invasive brain stimulation techniques, such as repetitive transcranial magnetic stimulation (rTMS) and transcranial direct current stimulation (tDCS), have emerged as powerful tools for modulating prefrontal activity. These techniques not only help elucidate the neural underpinnings of cognitive functions but also hold promise for treating psychiatric disorders such as depression and anxiety (Gershon et al. 2003, Perera et al. 2016, Polanía et al. 2018, Pitcher et al. 2021). Preliminary evidence highlights the causal role of the rDLPFC in memory control. For instance, low-frequency rTMS, which deactivates the rDLPFC, has been shown to impair DF performance (Xie et al. 2020), while disrupting prefrontal activity via tDCS similarly diminishes the DF effect (Silas and Brandt 2016, Imbernón et al. 2022). Moreover, our prior studies showed that healthy individuals can voluntarily forget negative social feedback (Chen et al. 2021, Xie et al. 2021), and high-frequency rTMS over the rDLPFC improves this ability in depressed patients (Chen et al. 2021). Yet, no study has directly compared the effects of brain stimulation on social versus non-social memories, leaving gaps in our understanding of social memory regulation and neuromodulation’s selective efficacy.

The present study aims to address this gap by investigating the causal role of rDLPFC stimulation in the voluntary forgetting of social versus non-social memories. High-frequency rTMS enhances cortical excitability via mechanisms akin to long-term potentiation, strengthening synaptic connections (Dayan et al. 2013, Klomjai et al. 2015) and top-down control when applied to DLPFC (Zhao et al. 2021, Asl and Vaghef 2022, Pulopulos et al. 2022). Using high­frequency (10 Hz) rTMS to enhance rDLPFC activity, we recruited participants to complete a DF task in either a social judgement context (framed as peer feedback) or a non-social context. By employing a mixed design, we hypothesized that rDLPFC stimulation would enhance DF performance for non-social memories but exert minimal effects on social memories, given the tendency for self-threatening social information to undergo spontaneous forgetting (Sedikides et al. 2016, Zengel et al. 2018, Rigney et al. 2021).

Additionally, we examined the moderating role of psychiatric symptoms, particularly social anxiety, on the effects of rDLPFC stimulation. Previous research suggests that impaired prefrontal control contributes to memory regulation difficulties in individuals with depression and social anxiety (Delaney et al. 2020, Costanzi et al. 2021, Stramaccia et al. 2021), who often struggle to forget distressing social memories (Saunders 2011, Zengel et al. 2015). We hypothesized that participants with higher social anxiety would exhibit greater improvements in social DF performance following rDLPFC stimulation. These findings could inform neuromodulation-based interventions for alleviating memory biases and emotional distress associated with social anxiety.

## Methods

### Participants

This study recruited two groups of participants: a non-social memory group and a social memory group. Based on prior TMS research in our lab using social feedback materials (Li et al. 2022), we initially aimed to recruit 40 participants per group to achieve adequate statistical power. To account for the possibility that some participants might not believe the social evaluative cover story (Nasso et al. 2022), a total of 90 healthy, right-handed college students from Shenzhen University were recruited—40 for the non-social group and 50 for the social group.

After post-experiment interview, eight participants in the social group were excluded due to disbelief in the cover story, resulting in a final sample of 82 participants. Sensitivity analyses conducted using G*Power 3.1 indicated that this sample size provided 80% statistical power to detect an effect size of *f* = 0.13 in a mixed design ANOVA, assuming a false positive rate of 5%.

In the non-social group (*n* = 40, 18 males), participants were aged 18 to 23 years (M ± SD = 19.7 ± 1.5). In the social group (*n* = 42, 23 males), participants were aged 18 to 25 years (20.2 ± 1.7). None of the participants had prior experience with TMS. Demographic characteristics for both groups are summarized in Table 1. The study was approved by the Ethics Committee of Shenzhen University. All participants provided written informed consent before participation and were monetarily compensated (60 CNY/hour).

**Table 1.** Demographic characteristics of the two groups (mean ± SD).

Items	Social group (*n* = 42)	nonsocial group (*n* = 40)	Statistics^a^	
			*t*_(80)_	*P*
Gender (male/female)	23/19	18/22		
Age (years)	20.2 ± 1.7	19.7 ± 1.5	1.381	.171
BDI-II	6.6 ± 5.1	5.9 ± 5.5	0.656	.514
STAI-T	40.8 ± 7.5	41.6 ± 8.4	−0.449	.655
LSAS	53.7 ± 18.5	49.1 ± 21.2	1.052	.232
RSQ	10.5 ± 2.6	10.8 ± 2.4	−0.489	.626
RSAS	9.9 ± 4.5	9.1 ± 5.0	0.813	.419

a Independent samples *t*-test between the two groups.

BDI-II, the Beck Depression Inventory Second Edition; STAI-T, the Trait form of Spielberger’s State-Trait Anxiety Inventory; LSAS, the Liebowitz Social Anxiety Scale; RSQ, the Rejection Sensitivity Questionnaire; RSAS, the Revised Social Anhedonia Scale.

### Experimental materials and study design

The directed forgetting (DF) task used 80 two-character adjectives (40 negative and 40 positive) selected from the Chinese Affective Words System (CAWS; Wang et al. 2008), which are commonly used to describe personality traits. Negative and positive words were counterbalanced across the four conditions (*TMS condition* × *DF cue*), with 10 words assigned to each condition. The word sets were balanced for valence and arousal ratings across conditions (*P*s > .05).

For the recognition test, an additional 80 adjectives describing personality traits were selected from the CAWS to serve as new items. There were no significant differences in valence and arousal between old and new word sets (*P*s > .05).

As in previous studies using a similar social evaluative cover story (Nasso et al. 2022; Xie et al. 2023), positive social feedback conditions were included to enhance the credibility of the cover story. However, since the study focused on the voluntary forgetting of negative memories, positive feedback conditions were excluded from the analyses. This resulted in a 2 (*Material group*: Social vs. Non-social) × 2 (*TMS condition*: rDLPFC-activated Active vs. vertex-­activated Control) × 2 (*DF cue*: TBR vs. TBF) mixed design. The two within-subject factors were *TMS condition* and *DF cue*, while the between-subject factor was *Material group*.

### Experimental procedure

**Figure 1.**
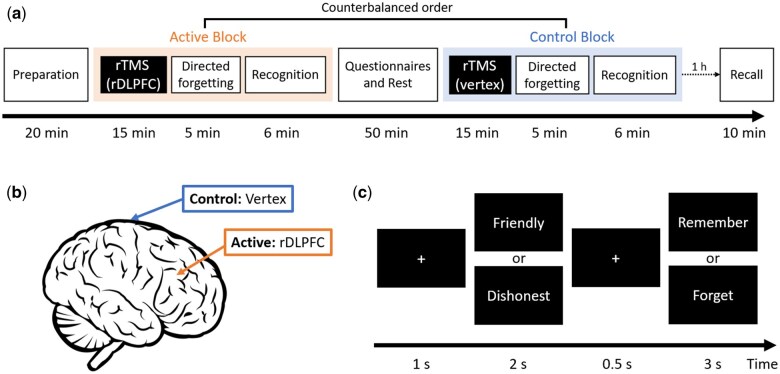
Experimental procedures. (a) Overview of the five phases of the experiment. (b) Stimulation positions for the two TMS conditions: the right dorsolateral prefrontal cortex (rDLPFC) for the Active and the vertex for Control condition. (c) Illustration of a single trial in the directed forgetting task.

The experiment consisted of five phases (Fig. 1a).

#### Phase 1: preparation stage

Participants in the social group were informed that the study aimed to examine brain activity during the processing of social feedback. Upon registration, participants provided an identity photo, which they were told would be evaluated by peers from a neighbouring university. They were informed that their peers had selected one of two opposite adjectives (e.g. “honest” vs. “dishonest”) to describe their first impression, and that these adjectives would be presented during the task (Somerville et al. 2006, Nasso et al. 2022). Participants were debriefed post-experiment to assess their belief in this cover story.

Participants in the non-social group were told that the study explored the relationship between brain activity and attentional control. Both groups received an introduction to the TMS equipment and procedures before the experiment began.

#### Phases 2 and 4: active and control blocks

In the active block, participants received 15 minutes of high­frequency (10 Hz) repetitive TMS (rTMS) stimulation over the rDLPFC. In the control block, they received identical stimulation over the vertex, serving as a control site (Fig. 1b). The order of the active and control blocks was counterbalanced across participants.

Following each stimulation session, participants completed a DF task. Each trial began with a 1-sesond fixation cross, followed by a positive or negative adjective presented for 2 s. A second fixation cross appeared for 0.5 s, after which a cue indicating “Remember” (“记”) or “Forget” (“忘”) was displayed for 3 s (Fig. 1c). The task included 40 trials per block, divided into four conditions: Positive-Remember, Positive-Forget, Negative-Remember, and Negative-Forget, with 10 trials per condition. Trial order was pseudo-randomized within blocks.

Participants then rested for 3 minutes before the old/new ­recognition task, where 40 old and 40 new words were randomly presented. Participants indicated within 2 s whether each word was old or new, followed by a 1-s blank screen. While many DF studies use interference tasks to prevent memory reconsolidation, we opted for a rest period to reduce cognitive fatigue due to the extended experimental session and TMS procedures (see also Shen et al. 2020). Though this approach may have allowed some memory reconsolidation, the focus on condition/group differences likely mitigates its impact on the main findings.

#### Phase 3: questionnaires and rest

Participants completed a battery of questionnaires, including: the Beck Depression Inventory-II (BDI-II; Beck et al. 1996), the State-Trait Anxiety Inventory—Trait Form (STAI-T; Spielberger et al. 1983), the Liebowitz Social Anxiety Scale (LSAS; Liebowitz 1987), the Rejection Sensitivity Questionnaire (RSQ; Downey and Feldman 1996), and the Revised Social Anhedonia Scale (RSAS; Eckblad et al. 1982). The questionnaire phase lasted approximately 50 minutes, allowing participants a rest period to minimize carryover effects between TMS sessions.

#### Phase 5: free recall task

Approximately one hour after completing the final recognition task, participants were asked to recall as many words as possible from the DF task, regardless of their associated cue type (TBR or TBF). They had 10 minutes to write down the recalled words, which were subsequently scored for accuracy. This free recall task, widely used in DF studies, measures memory accessibility and intentional forgetting efficacy (Bjork 1989, Basden and Basden 2013). This task is particularly sensitive to memory suppression effects, as it tests participants’ ability to voluntarily retrieve or inhibit memories, making it valuable for studying the mechanisms of intentional forgetting.

### Repetitive transcranial magnetic stimulation

An offline TMS protocol was used to minimize potential side effects that could affect task performance. In the Active condition, rTMS targeted the rDLPFC, while the vertex was chosen as the control site. The vertex was selected because stimulating this area induces a similar scalp sensation to the Active condition (Zhao et al. 2021, Li et al. 2022).

A figure-of-eight coil connected to a magnetic stimulator (M-100 Ultimate; Yingchi, Shenzhen, China) was used to deliver stimulation pulses. Coil placement was determined based on the International 10/20 EEG system, with the right DLPFC corresponding to the F4 site and the vertex corresponding to the Cz site (Zhao et al. 2021, Li et al. 2022). Resting motor thresholds (rMT) were measured at the C3 site.

Stimulation was delivered at 10 Hz, 90% of the participant’s rMT (Lefaucheur et al. 2008, Park et al. 2017, Li et al. 2022). Each session lasted 15 minutes, comprising 30 trains of 4-s stimulation with 26-s inter-train intervals. In total, each session delivered 1200 pulses. The 10-Hz frequency was selected for its excitatory effects, promoting long-term potentiation in the targeted brain region (Dayan et al. 2013). High-frequency rTMS effects can persist beyond the stimulation period (Dayan et al. 2013, Klomjai et al. 2015), ensuring coverage throughout the 5-minute directed forgetting task.

### Statistical analysis

Statistical analyses were performed using jamovi 1.0.7.0 (https://www.jamovi.org). Descriptive data are reported as Mean ± SD, unless otherwise specified.

Repeated-measures ANOVAs were conducted to assess task performance. Within-subject factors were *TMS condition* (Active vs. Control) and *DF cue* (TBR vs. TBF), and the between-subject factor was *Material group* (Social vs. Non-social).

To explore relationships between self-reported measures and task performance, two-tailed Pearson’s correlations were conducted between questionnaire scores (BDI-II, STAI-T, LSAS, RSQ, and RSAS) and behavioural indicators (hit rate, false alarms, recognition *d’*, and recall accuracy) separately for each group. Due to the exploratory nature, correlations were not corrected for multiple comparisons.

## Results

For clarity, descriptive data (Mean ± SD) for all measured variables, excluding false alarms, are presented in Table 2.

**Table 2.** Mean ± SD of each within-subject condition in the social and nonsocial group.

	Social (*n *= 42)				Nonsocial (*n *= 40)			
	TBR		TBF		TBR		TBF	
	Active	Control	Active	Control	Active	Control	Active	Control
Hit rate	0.76 ± 0.17	0.77 ± 0.15	0.62 ± 0.17	0.64 ± 0.21	0.83 ± 0.15	0.77 ± 0.18	0.52 ± 0.21	0.63 ± 0.20
Sensitivity (*d’*)	1.51 ± 0.88	1.43 ± 0.72	0.98 ± 0.54	0.97 ± 0.76	1.82 ± 0.88	1.63 ± 0.87	0.65 ± 0.60	1.04 ± 0.63
Recall accuracy	0.30 ± 0.14	0.34 ± 0.19	0.09 ± 0.10	0.10 ± 0.13	0.31 ± 0.17	0.26 ± 0.19	0.06 ± 0.09	0.06 ± 0.08

### Recognition performance

Participants’ hit rates (Hit) and false alarm rates (FA) were calculated for each condition. Noted that TBR and TBF items were intermixed with a common set of new items during each recognition task, there were four FA conditions: Social-Active (0.30 ± 0.18), Social-Control (0.31 ± 0.17), Non-social-Active (0.30 ± 0.19), and Non-social-Control (0.28 ± 0.17).

#### Hit rates

A repeated-measures ANOVA revealed a significant main effect of *DF cue* (*F*(1,80) = 103.881, *P *< .001, $\eta_{p}^{2\;}$ = 0.565), with higher hit rates for TBR items compared to TBF items. Additionally, a two-way ­interaction between *DF cue* and *TMS condition* was observed (*F*(1,80) = 6.985, *P *= .010, $\eta_{p}^{2\;}$ = 0.080). Simple effects analysis indicated that rDLPFC activation reduced hit rates for TBF items compared to the Control condition (*P *= .004), but had no effect on TBR items (*P *= .339). Besides, a two-way interaction was found between *DF cue* and *Material group* (*F*(1,80) = 6.419, *P *= .013, $\eta_{p}^{2\;}$= 0.074). Simple effects analysis revealed that participants in the Social group showed a trend towards higher hit rates for TBF items compared to the Non-social group (*P *= .088), whereas hit rates for TBR items were comparable across groups (*P *= .333). Furthermore, both the Social and Non-social groups showed significant DF effects, with higher hit rate for TBR items compared to TBF items (*P*s < .001). However, the magnitude of the DF effect (TBR minus TBF) was smaller for the social group (mean difference = 0.139) than for the non-social group (mean difference = 0.230).

Moreover, a significant three-way interaction was found (*F*(1,80) = 6.745, *P = *.011, $\eta_{p}^{2\;}$ = 0.078). To break down this three-way interaction, we tested the *DF* × *TMS* interaction separately for each group. Results showed that this two-way interaction was significant in the Non-social group (*F*(1,39) = 13.515, *P < *.001, $\eta_{p}^{2\;}$ = 0.257) but not in the Social group (*F*(1,41) = 0.001, *P = *.974, $\eta_{p}^{2\;}$ = 0.000). Specifically, in the Non-social group, active TMS reduced hit rates for TBF items compared to the control TMS condition (*P *< .001) and showed a trend towards improving hit rates for TBR items (*P *= .074).

In addition, in the Social group, participants’ social anxiety scores were positively correlated with hit rates in the TBR-Control (*r *= 0.354, *P *= .021) and TBF-Control (*r *= 0.338, *P *= .029) conditions. However, these correlations disappeared in the TBR-Active (*r *= 0.234, *P *= .135) and TBF-Active (*r *= 0.206, *P *= .192) conditions.

#### False alarms

A repeated-measures ANOVA yielded no significant main effects or interactions across conditions.

#### Recognition sensitivity (*d’*)

Recognition sensitivity (*d’*) was calculated using the formula: *d’* = z(Hit) − z(FA) (Macmillan and Creelman 1991). Higher *d’* values reflect better discrimination between old and new items. Given the lack of significant FA effects, *d’* patterns were primarily driven by differences in hit rates.

A significant main effect of *DF cue* was found (*F*(1,80) = 113.466, *P *< .001, $\eta_{p}^{2\;}$ = 0.586), with TBR items recognized better than TBF items, consistent with the DF effect (Bjork 1989, Anderson and Hanslmayr 2014). Furthermore, a two-way interaction between *DF cue* and *TMS condition* was observed (*F*(1,80) = 7.581, *P *= .007, $\eta_{p}^{2\;}$ = 0.087). Active TMS tended to reduce recognition of TBF items compared to the Control (*P *= .061) but did not affect recognition of TBR items (*P *= .181). A two-way interaction between *DF cue* and *Material group* was found (*F*(1,80) = 9.110, *P *= .003, $\eta_{p}^{2\;}$ = 0.102). Participants in the Social group showed poorer recognition for TBR items compared to the Non-social group (*P *= .048), whereas recognition for TBF items was comparable (*P *= .322).

**Figure 2.**
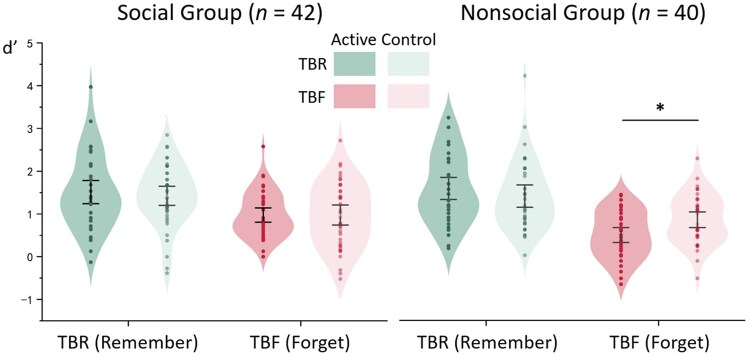
Recognition sensitivity (*d’*) results. A significant three-way interaction was found between the DF cue, TMS condition, and Material group. Post-hoc analyses revealed a significant TMS effect on the TBF condition in the Nonsocial group. Bars represent the standard error of the mean. *P < .05.

The key finding was the significant three-way interaction (*F*(1,80) = 4.380, *P = *.040, $\eta_{p}^{2\;}$ = 0.052; see Fig. 2). To further explore this interaction, we examined the *DF cue* × *TMS condition* interaction within each group. The results revealed that this two-way interaction was significant in the Non-social group (*F*(1,39) = 10.725, *P = *.002, $\eta_{p}^{2\;}$ = 0.216), but not in the Social group (*F*(1,41) = 0.239, *P = *.627, $\eta_{p}^{2\;}$ = 0.006). Specifically, in the Non-social group, Active TMS reduced recognition sensitivity for TBF items (*P *= .013) but not affect TBR items (*P *= .224).

Additionally, in the social group, *d’* scores in the TBF-Control condition were positively correlated with social anxiety scores (*r *= 0.313, *P *= .044), indicating that higher social anxiety was associated with reduced ability to forget negative social feedback. However, this correlation disappeared in the TBF-Active condition (*r *= −0.215, *P *= .171). Besides, no significant correlations was found between social anxiety and TBR conditions (Control: *r *= 0.258, *P *= .099; Active: *r *= −0.035, *P *= .827).

**Figure 3.**
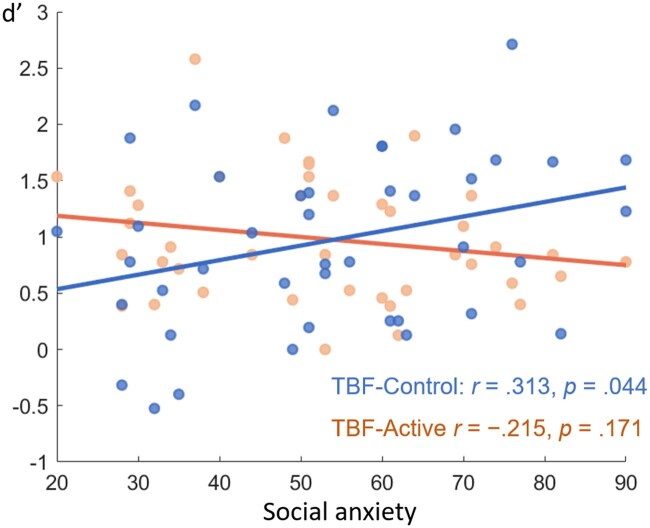
Transcranial magnetic stimulation moderates the relationship between social anxiety and recognition performance of negative social feedback.

To further explore the moderating role of social anxiety (SA) on the TMS effect within the Social Group, we conducted a multiple regression analysis with recognition sensitivity (*d’*) as the dependent variable. The predictors included DF (TBR vs. TBF), TMS (Active vs. Control), SA level, and their interactions (DF×TMS, DF × SA, TMS × SA, and DF ×TMS × SA). This analysis was performed using the “lm” function in R, which fits linear models to the data (Montgomery et al. 2021). The multiple regression analysis (*F*(7, 160) = 3.994, *P* < .001, adjusted *R*^2^ = 0.112) revealed a significant main effect of TMS (*β* = 1.036, *P* = .038), a significant main effect of SA (*β* = 0.013, *P* = .038), and a significant TMS × SA interaction (*β *= 0.62, *P* = .043). Other main effects and interactions were not significant. These findings suggest that social anxiety moderates the effect of TMS on memories of social feedback. Conversely, TMS also moderates the relationship between social anxiety and memories of social feedback (Fig. 3).

We also examined the impact of TMS on the DF effect of negative social feedback in participants who scored above 60 on the Liebowitz Social Anxiety Scale (*N* = 18). A repeated-measures ANOVA revealed a significant main effect of *DF cue* (*F*(1,17) = 12.02, *P *= .003, $\eta_{p}^{2\;}$ = 0.414), with higher recognition sensitivity for TBR items compared to TBF items. Additionally, the ANOVA revealed a trend towards a two-way interaction between *DF cue* and *TMS condition* (*F*(1,17) = 2.18, *P *= .159, $\eta_{p}^{2\;}$ = 0.113). Simple effects analysis indicated that rDLPFC activation reduced recognition sensitivity for TBF items compared to the Control condition (*P *= .043), but had no effect on TBR items (*P *= .797).

### Recall performance

The ANOVA revealed a significant main effect of *DF cue* (*F*(1,80) = 231.522, *P *< .001, $\eta_{p}^{2\;}$ = 0.743), with participants recalling more TBR items than TBF items.

Correlation analyses indicated that, in the social group, recall accuracy was positively correlated with social anhedonia scores in the TBF-Control condition (*r *= 0.367, *P *= .017), but this correlation disappeared in the TBF-Active condition (*r *= −0.047, *P *= .768). Additionally, recall accuracy was negatively correlated with rejection sensitivity scores in the TBF-Active condition (*r *= −0.308, *P *= .047), but not in the TBF-Control condition (*r *= −0.064, *P *= .686).

To enhance the comprehensiveness of the findings, supplementary analyses on memory performance (including recognition sensitivity and recall) under positive conditions have been conducted and reported in the Supplementary Materials.

## Discussion

The ability to voluntarily forget unwanted memories is essential for mental well-being, enabling individuals to discard information that disrupts focus or exacerbates emotional distress (Hu et al. 2017, Engen and Anderson 2018). This study used a directed forgetting (DF) paradigm and repetitive transcranial magnetic stimulation (rTMS) to investigate the causal role of the right dorsolateral prefrontal cortex (rDLPFC) in the voluntary forgetting of social and non-social memories. By contrasting the effects of rTMS in social and non-social contexts, our findings reveal distinct neural mechanisms underlying the regulation of these memory types and highlight the potential therapeutic applications of prefrontal stimulation for individuals with social anxiety.

### DLPFC and voluntary forgetting of non-social memories

Consistent with prior research, the rDLPFC was found to play a central role in the voluntary forgetting of non-social memories (Wylie et al. 2008, Nowicka et al. 2011; see Anderson and Hanslmayr 2014 for a review), likely via top-down inhibitory control over hippocampal activity (Rizio and Dennis 2013, Oehrn et al. 2018, Anderson and Hulbert 2021, Hubbard and Sahakyan 2023). Our previous work has shown that disrupting rDLPFC activity impairs DF performance (Xie et al. 2020); this study extends these findings by demonstrating that high-frequency rTMS enhances voluntary forgetting of non-social memories. This effect was specific to to-be-forgotten (TBF) items, as recognition of to-be-remembered (TBR) items remained intact. High-frequency rTMS likely strengthens top-down control via excitatory effects on synaptic plasticity (Dayan et al. 2013, Klomjai et al. 2015).

These results align with theoretical models emphasizing the selective inhibitory function of the rDLPFC in memory control, allowing individuals to suppress irrelevant or undesired information while preserving relevant content (Anderson and Hanslmayr 2014, Anderson and Hulbert 2021). The findings further underscore the adaptability of prefrontal inhibitory processes in regulating non-social memories, which rely heavily on top-down modulation.

### Social memories and reduced dependence on DLPFC control

In contrast, the voluntary forgetting of social memories appeared less dependent on rDLPFC-mediated inhibition. Participants in the social memory group showed no significant enhancement of DF performance following rDLPFC stimulation. This may reflect the automatic suppression of self-threatening social feedback during encoding, reducing reliance on prefrontal control mechanisms for active forgetting (Xie et al. 2021). Supporting this interpretation, participants in the social memory group displayed poorer recognition of TBR items compared to the non-social group, consistent with reduced encoding of self-threatening social information (Zengel et al. 2018, Rigney et al. 2021).

The lack of TBF memory differences between groups may stem from non-social memories being more susceptible to intentional forgetting than spontaneous forgetting, potentially masking group effects. This suggests that social memory regulation involves alternative or differently weighted neural mechanisms compared to non-social memory, with less reliance on prefrontal inhibition.

### Social anxiety and forgetting of negative social feedback

A key finding was that rTMS moderated the link between social anxiety and negative social memory. In the Control TMS condition, participants with higher social anxiety showed greater difficulty in forgetting TBF social items, consistent with previous research linking social anxiety to impaired voluntary forgetting (Gomez-Ariza et al. 2013). However, this impairment was alleviated under Active TMS, suggesting that rDLPFC stimulation can mitigate memory biases associated with social anxiety.

Individuals with social anxiety often struggle with spontaneous forgetting of self-threatening memories, leading to persistent emotional distress (Zengel et al. 2015). By enhancing rDLPFC activity, rTMS may strengthen voluntary forgetting mechanisms, helping to counteract these deficits. This finding has clinical implications, as maladaptive retention of negative social memories is a key cognitive feature of social anxiety disorder (SAD; Coles and Heimberg 2002, Seinsche et al. 2023, Fricke et al. 2024). Prefrontal stimulation may thus represent a promising intervention for alleviating cognitive and emotional burdens in socially anxious individuals, particularly when combined with behavioural therapies targeting memory biases (Morgan 2010, Krans et al. 2014, Jarcho et al. 2015, Gong et al. 2023).

### Discrepancy between recall and recognition performance

Interestingly, recall performance did not mirror recognition results, with no observed TMS or material effects. This discrepancy may reflect differences in retrieval processes: recall relies on active memory generation, while recognition involves cue-driven retrieval. The rDLPFC’s impact on non-social memory may be tied to familiarity-based processes relevant to recognition tasks (Rugg and Yonelinas 2003). Additionally, the delay between TMS and the recall task could have diminished rTMS effects, given their transient nature (Dayan et al. 2013, Klomjai et al. 2015).

### Limitations and future directions

This study has several limitations that warrant consideration. The between-subject design may have introduced unmeasured confounds, such as individual differences in baseline memory capacity or task engagement, which could be addressed in future studies using within-subject designs to provide stronger evidence for context-dependent effects. Additionally, the lack of neuroimaging data limits our ability to directly link behavioural outcomes to neural activity. Incorporating fMRI-guided neuronavigation (Polanía et al. 2018, Pitcher et al. 2021) would enhance TMS targeting precision and clarify the neural mechanisms underlying memory regulation. The limited sample size, particularly in subgroups such as participants with high social anxiety, may have reduced the power to detect individual differences. Future research with larger, more balanced samples is needed to draw definitive conclusions. Moreover, the absence of a baseline condition makes it challenging to disentangle intentional forgetting from spontaneous suppression processes. Incorporating a baseline condition, similar to the Think/No-Think paradigm (Anderson and Green 2001, Anderson and Hanslmayr 2014), would enable a more rigorous investigation of directed forgetting effects. Finally, placing the questionnaire phase between the two DF tasks may have introduced confounding effects due to task order. However, given that the questionnaires primarily measured stable trait-like characteristics such as social anxiety and rejection sensitivity, their influence on the results is likely minimal. To mitigate any potential interference, future studies should administer questionnaires at the outset of the experiment.

### Conclusion

This study provides novel evidence of a dissociation in the neural mechanisms underlying the voluntary forgetting of social and non-social memories. While rDLPFC stimulation enhanced DF performance for non-social memories, it had no significant effect on social memories, likely due to their reliance on automatic encoding biases rather than prefrontal inhibitory control. Importantly, rTMS over the rDLPFC mitigated memory biases in individuals with high social anxiety, offering a potential avenue for targeted interventions. These findings deepen our understanding of the neural basis of memory control and suggest innovative strategies for addressing maladaptive memory retention in clinical populations.

## Supplementary Material

nsaf052_Supplementary_Data

## Data Availability

The data and code of this study would be available upon reasonable request and with the approval of the School of Psychology, Shenzhen University. More information on making this request can be obtained from the corresponding author D. Zhang (zhangdd05@gmail.com).

## References

[nsaf052-B1] Anderson MC , Green C. Suppressing unwanted memories by executive control. Nature 2001;410:366–9. 11268212 10.1038/35066572

[nsaf052-B2] Anderson MC , Hanslmayr S. Neural mechanisms of motivated forgetting. Trends Cogn Sci 2014;18:279–92. 24747000 10.1016/j.tics.2014.03.002 PMC4045208

[nsaf052-B3] Anderson MC , Hulbert JC. Active forgetting: adaptation of memory by prefrontal control. Annu Rev Psychol 2021;72:1–36. 32928060 10.1146/annurev-psych-072720-094140

[nsaf052-B4] Asl FA , Vaghef L. The effectiveness of high-frequency left DLPFC-rTMS on depression, response inhibition, and cognitive flexibility in female subjects with major depressive disorder. J Psychiatr Res 2022;149:287–92. 35313201 10.1016/j.jpsychires.2022.01.025

[nsaf052-B6] Basden BH , Basden DR. Directed forgetting: a contrast of methods and interpretations. In: Intentional Forgetting. New York, USA: Psychology Press, 2013, 151–84.

[nsaf052-B7] Beck AT , Steer RA, Brown GK. Beck Depression Inventory, 2nd ed. San Antonio, Texas, USA: The Psychological Corporation, 1996.

[nsaf052-B8] Bjork RA. Retrieval inhibition as an adaptive mechanism in human memory. In: Roediger HL, Craik FIM (eds.), Varieties of Memory and Consciousness: Essays in Honour of Endel Tulving. Hillsdale, New Jersey, USA: Erlbaum, 1989, 309–30

[nsaf052-B9] Chen Y , Li S, Guo T et al The role of dorsolateral prefrontal cortex on voluntary forgetting of negative social feedback in depressed patients: a TMS study. Acta Psychol Sin 2021;53:1094–104.

[nsaf052-B10] Coles ME , Heimberg RG. Memory biases in the anxiety disorders: current status. Clin Psychol Rev 2002;22:587–627. 12094512 10.1016/s0272-7358(01)00113-1

[nsaf052-B11] Costanzi M , Cianfanelli B, Santirocchi A et al Forgetting unwanted memories: active forgetting and implications for the development of psychological disorders. J Pers Med 2021;11:241. 33810436 10.3390/jpm11040241 PMC8066077

[nsaf052-B12] Dayan E , Censor N, Buch ER et al Noninvasive brain stimulation: from physiology to network dynamics and back. Nat Neurosci 2013;16:838–44. 23799477 10.1038/nn.3422 PMC4876726

[nsaf052-B13] Delaney PF , Barden EP, Smith WG et al What can directed forgetting tell us about clinical populations? Clin Psychol Rev 2020;82:101926. 33011552 10.1016/j.cpr.2020.101926

[nsaf052-B15] Downey G , Feldman SI. Implications of rejection sensitivity for intimate relationships. J Pers Soc Psychol 1996;70:1327–43. 8667172 10.1037//0022-3514.70.6.1327

[nsaf052-B16] Eckblad ML , Chapman LJ, Chapman JP et al The revised social anhedonia scale. Unpublished manuscript, University of Wisconsin - Madison.

[nsaf052-B17] Einarsen S , Mikkelsen EG. Individual effects of exposure to bullying at work. In: Bullying and Emotional Abuse in the Workplace. Boca Raton, Florida, USA: CRC Press, 2002, 145–62

[nsaf052-B18] Engen HG , Anderson MC. Memory control: a fundamental mechanism of emotion regulation. Trends Cogn Sci 2018;22:982–95. 30122359 10.1016/j.tics.2018.07.015 PMC6198111

[nsaf052-B19] Fawcett JM , Taylor TL, Megla E et al Active intentional and unintentional forgetting in the laboratory and everyday life. Nat Rev Psychol 2024;3:652–64.

[nsaf052-B20] Fricke S , Seinsche RJ, Neudert MK et al Neural correlates of context­dependent extinction recall in social anxiety disorder: relevance of intrusions in response to aversive social experiences. Psychol Med 2024;54:548–57. 37553977 10.1017/S0033291723002179

[nsaf052-B21] Fung K , Alden LE. Once hurt, twice shy: social pain contributes to social anxiety. Emotion 2017;17:231–9. 27606825 10.1037/emo0000223

[nsaf052-B22] Gamboa OL , Sung Lai Yuen K, von Wegner F et al The challenge of forgetting: neurobiological mechanisms of auditory directed forgetting. Hum Brain Mapp 2018;39:249–63. 29080232 10.1002/hbm.23840 PMC6866323

[nsaf052-B23] Gershon AA , Dannon PN, Grunhaus L. Transcranial magnetic stimulation in the treatment of depression. Am J Psychiatry 2003;160:835–45. 12727683 10.1176/appi.ajp.160.5.835

[nsaf052-B24] Gomez-Ariza CJ , Iglesias-Parro S, Garcia-Lopez LJ et al Selective intentional forgetting in adolescents with social anxiety disorder. Psychiatry Res 2013;208:151–5. 23068080 10.1016/j.psychres.2012.09.027

[nsaf052-B25] Gong H , Cao Q, Li M. Social memory characteristics of non-clinical college students with social anxiety. J Affect Disord 2023;326:147–54. 36708955 10.1016/j.jad.2023.01.075

[nsaf052-B27] Hu X , Bergström ZM, Gagnepain P et al Suppressing unwanted memories reduces their unintended influences. Curr Dir Psychol Sci 2017;26:197–206. 28458471 10.1177/0963721417689881 PMC5390940

[nsaf052-B28] Hubbard RJ , Sahakyan L. Differential recruitment of inhibitory control processes by directed forgetting and thought substitution. J Neurosci 2023;43:1963–75. 36810228 10.1523/JNEUROSCI.0696-22.2023 PMC10027038

[nsaf052-B29] Imbernón JJ , Aguirre C, Gómez-Ariza CJ. Selective directed forgetting is mediated by the lateral prefrontal cortex: preliminary evidence with transcranial direct current stimulation. Cogn Neurosci 2022;13:77–86. 34283693 10.1080/17588928.2021.1953973

[nsaf052-B30] Jarcho JM , Romer AL, Shechner T et al Forgetting the best when predicting the worst: preliminary observations on neural circuit function in adolescent social anxiety. Dev Cogn Neurosci 2015;13:21–31. 25933410 10.1016/j.dcn.2015.03.002 PMC4466042

[nsaf052-B31] Klomjai W , Katz R, Lackmy-Vallée A. Basic principles of transcranial magnetic stimulation (TMS) and repetitive TMS (rTMS). Ann Phys Rehabil Med 2015;58:208–13. 26319963 10.1016/j.rehab.2015.05.005

[nsaf052-B32] Krans J , de Bree J, Bryant RA. Autobiographical memory bias in social anxiety. Memory 2014;22:890–7. 24111655 10.1080/09658211.2013.844261

[nsaf052-B35] Lefaucheur JP , Drouot X, Menard-Lefaucheur I et al Motor cortex rTMS in chronic neuropathic pain: pain relief is associated with thermal sensory perception improvement. J Neurol Neurosurg Psychiatry 2008;79:1044–9. 18223016 10.1136/jnnp.2007.135327

[nsaf052-B36] Levy BJ , Anderson MC. Purging of memories from conscious awareness tracked in the human brain. J Neurosci 2012;32:16785–94. 23175832 10.1523/JNEUROSCI.2640-12.2012 PMC3544307

[nsaf052-B38] Li S , Xie H, Zheng Z et al The causal role of the bilateral ventrolateral prefrontal cortices on emotion regulation of social feedback. Hum Brain Mapp 2022;43:2898–910. 35261115 10.1002/hbm.25824 PMC9120569

[nsaf052-B39] Liebowitz MR. Social phobia. Mod Probl Pharmacopsychiatry 1987;22:141–73. 2885745 10.1159/000414022

[nsaf052-B40] Macmillan NA , Creelman BD. Detection Theory: A User’s Guide. New York: Bambridge University Press, 1991.

[nsaf052-B42] Mary A , Dayan J, Leone G et al Resilience after trauma: the role of memory suppression. Science 2020;367:eaay8477. 32054733 10.1126/science.aay8477

[nsaf052-B43] Montgomery DC , Peck EA, Vining GG. Introduction to Linear Regression Analysis. Hoboken, New Jersey, USA: John Wiley & Sons, 2021.

[nsaf052-B44] Morgan J. Autobiographical memory biases in social anxiety. Clin Psychol Rev 2010;30:288–97. 20067854 10.1016/j.cpr.2009.12.003

[nsaf052-B45] Nasso S , Vanderhasselt MA, Schettino A et al The role of cognitive reappraisal and expectations in dealing with social feedback. Emotion 2022;22:982–91. 32881546 10.1037/emo0000825

[nsaf052-B46] Nørby S. Why forget? On the adaptive value of memory loss. Perspect Psychol Sci 2015;10:551–78. 26385996 10.1177/1745691615596787

[nsaf052-B47] Nørby S. Forgetting and emotion regulation in mental health, anxiety and depression. Memory 2018;26:342–63. 28697639 10.1080/09658211.2017.1346130

[nsaf052-B48] Nowicka A , Marchewka A, Jednoróg K et al Forgetting of emotional information is hard: an fMRI study of directed forgetting. Cereb Cortex 2011;21:539–49. 20584747 10.1093/cercor/bhq117

[nsaf052-B49] Oehrn CR , Fell J, Baumann C et al Direct electrophysiological evidence for prefrontal control of hippocampal processing during voluntary forgetting. Curr Biol 2018;28:3016–22.e4. 30197086 10.1016/j.cub.2018.07.042

[nsaf052-B50] Park E , Kim MS, Chang WH et al Effects of bilateral repetitive transcranial magnetic stimulation on post-stroke dysphagia. Brain Stimul 2017;10:75–82. 27593709 10.1016/j.brs.2016.08.005

[nsaf052-B51] Perera T , George MS, Grammer G et al The clinical TMS society consensus review and treatment recommendations for TMS therapy for major depressive disorder. Brain Stimul 2016;9:336–46. 27090022 10.1016/j.brs.2016.03.010 PMC5612370

[nsaf052-B52] Pitcher D , Parkin B, Walsh V. Transcranial magnetic stimulation and the understanding of behavior. Annu Rev Psychol 2021;72:97–121. 33095690 10.1146/annurev-psych-081120-013144

[nsaf052-B53] Polanía R , Nitsche MA, Ruff CC. Studying and modifying brain function with non-invasive brain stimulation. Nat Neurosci 2018;21:174–87. 29311747 10.1038/s41593-017-0054-4

[nsaf052-B54] Pulopulos MM , Allaert J, Vanderhasselt MA et al Effects of HF-rTMS over the left and right DLPFC on proactive and reactive cognitive control. Soc Cogn Affect Neurosci 2022;17:109–19. 32613224 10.1093/scan/nsaa082 PMC8824550

[nsaf052-B55] Rappaport BI , Barch DM. Brain responses to social feedback in internalizing disorders: a comprehensive review. Neurosci Biobehav Rev 2020;118:784–808. 32956691 10.1016/j.neubiorev.2020.09.012 PMC12403867

[nsaf052-B56] Rigney AE , Schnyer DM, Hu X et al Mechanisms of a spotless self-­image: navigating negative, self-relevant feedback. Self Identity 2021;20:1057–76.

[nsaf052-B57] Rizio AA , Dennis NA. The neural correlates of cognitive control: successful remembering and intentional forgetting. J Cogn Neurosci 2013;25:297–312. 23066730 10.1162/jocn_a_00310

[nsaf052-B58] Rohde KB , Caspar F, Koenig T et al Neurophysiological traces of interpersonal pain: how emotional autobiographical memories affect event-related potentials. Emotion 2018;18:290–303. 28857583 10.1037/emo0000356

[nsaf052-B59] Rugg MD , Yonelinas AP. Human recognition memory: a cognitive neuroscience perspective. Trends Cogn Sci 2003;7:313–9. 12860190 10.1016/s1364-6613(03)00131-1

[nsaf052-B60] Saunders J. Reversed mnemic neglect of self-threatening memories in dysphoria. Cogn Emot 2011;25:854–67. 21824025 10.1080/02699931.2010.524037

[nsaf052-B61] Sedikides C , Green JD, Saunders J et al Mnemic neglect: selective amnesia of one’s faults. Eur Rev Soc Psychol 2016;27:1–62.

[nsaf052-B62] Seinsche RJ , Fricke S, Neudert MK et al Memory representation of aversive social experiences in social anxiety disorder. J Anxiety Disord 2023;94:102669. 36669276 10.1016/j.janxdis.2023.102669

[nsaf052-B63] Shen W , Liu Z, Ball LJ et al Easy to remember, easy to forget? The memorability of creative advertisements. Creat Res J 2020;32:313–22.

[nsaf052-B64] Silas J , Brandt KR. Frontal transcranial direct current stimulation (tDCS) abolishes list-method directed forgetting. Neurosci Lett 2016;616:166–9. 26820374 10.1016/j.neulet.2016.01.035

[nsaf052-B65] Somerville LH , Heatherton TF, Kelley WM. Anterior cingulate cortex responds differentially to expectancy violation and social rejection. Nat Neurosci 2006;9:1007–8. 16819523 10.1038/nn1728

[nsaf052-B66] Spielberger CD , Gorsuch RL, Lushene RE, Vagg PR, Jacobs GA. Manual for the State-Trait Anxiety Inventory (Form Y1—Y2). Palo Alto, California, USA: Consulting Psychologist Press, 1983.

[nsaf052-B67] Stramaccia DF , Meyer AK, Rischer KM et al Memory suppression and its deficiency in psychological disorders: a focused meta-analysis. J Exp Psychol Gen 2021;150:828–50. 33090824 10.1037/xge0000971

[nsaf052-B68] Wang YN , Zhou LM, Luo YJ. The pilot establishment and evaluation of Chinese affective words system. Chin Ment Health J 2008;22:608–12.

[nsaf052-B69] Wylie GR , Foxe JJ, Taylor TL. Forgetting as an active process: an fMRI investigation of item-method directed forgetting. Cereb Cortex 2008;18:670–82. 17617657 10.1093/cercor/bhm101

[nsaf052-B70] Xie H , Chen Y, Lin Y et al Can’t forget: disruption of the right prefrontal cortex impairs voluntary forgetting in a recognition test. Memory 2020;28:60–9. 31645199 10.1080/09658211.2019.1681456

[nsaf052-B71] Xie H , Hu X, Mo L et al Forgetting positive social feedback is difficult: ERP evidence in a directed forgetting paradigm. Psychophysiology 2021;58:e13790. 33569800 10.1111/psyp.13790

[nsaf052-B72] Xie H , Lin X, Hu W et al Emotion regulation promotes forgetting of negative social feedback: behavioral and EEG evidence. Acta Psychologica Sinica 2023;55:905–19.

[nsaf052-B73] Yang W , Chen Q, Liu P et al Abnormal brain activation during directed forgetting of negative memory in depressed patients. J Affect Disord 2016;190:880–8. 26639452 10.1016/j.jad.2015.05.034

[nsaf052-B74] Yao Z , Lin X, Hu X. Optimistic amnesia: how online and offline processing shape belief updating and memory biases in immediate and long-term optimism biases. Soc Cogn Affect Neurosci 2021;16:453–62. 33502507 10.1093/scan/nsab011 PMC8094997

[nsaf052-B75] Zengel B , Skowronski JJ, Valentiner DP et al Loss of mnemic neglect among socially anxious individuals. J Soc Clin Psychol 2015;34:322–47.

[nsaf052-B76] Zengel B , Wells BM, Skowronski JJ. The waxing and waning of mnemic neglect. J Pers Soc Psychol 2018;114:719–34. 29672104 10.1037/pspa0000124

[nsaf052-B77] Zhao J , Mo L, Bi R et al The VLPFC versus the DLPFC in downregulating social pain using reappraisal and distraction strategies. J Neurosci 2021;41:1331–9. 33443069 10.1523/JNEUROSCI.1906-20.2020 PMC7888223

