# Supplementary material for: Targeting the dorsolateral prefrontal cortex to enhance memory control: divergent effects on social and non-social memories
Source: Soc Cogn Affect Neurosci. 2025 May 15;20(1):nsaf052. doi: 10.1093/scan/nsaf052 (PMC12322311; doi:10.1093/scan/nsaf052)
Supplement: nsaf052_Supplementary_Data [file nsaf052_supplementary_data.docx]

**Supplementary Materials**

For clarity, descriptive data (Mean ± SD) for recognition sensitivity (d’) and recall accuracy of positive-valence conditions are presented in Table S1.

**Recognition Performance for positive-valence conditions**

***Recognition Sensitivity (d’).*** A significant main effect of *DF cue* was found (*F*(1,80) = 147.606, *p* < 0.001, $\eta_{p}^{2}$= 0.649), with TBR items recognized better than TBF items. Furthermore, a two-way interaction between *DF cue* and *TMS condition* was observed (*F*(1,80) = 5.025, *p* = 0.028, $\eta_{p}^{2}$= 0.059). Active TMS reduced recognition of TBF items compared to the Control (*p* = 0.026) but did not affect recognition of TBR items (*p* = 0.754).

Furthermore, a significant three-way interaction was found (*F*(1,80) = 4.365, *p =* 0.040, $\eta_{p}^{2}$= 0.052). To further explore this interaction, we examined the *DF cue* × *TMS condition* interaction within each group. The results revealed that this two-way interaction was significant in the Nonsocial group (*F*(1,39) = 8.238, *p =* 0.007, $\eta_{p}^{2}$= 0.174), but not in the Social group (*F*(1,41) = 0.014, *p =* 0.909, $\eta_{p}^{2}$= 0.000). Specifically, in the Nonsocial group, Active TMS showed a trend to reduce recognition sensitivity for TBF items (*p* = 0.065), but this pattern was not shown for TBR items (*p* = 0.168).

**Recall Performance**

The ANOVA revealed a significant main effect of *DF cue* (*F*(1,80) = 186.938, *p* < 0.001, $\eta_{p}^{2}$= 0.700), with participants recalling more TBR items than TBF items. Furthermore, the *DF cue* × *Material group* interaction was significant (*F*(1,80) = 7.388, *p* = 0.008, $\eta_{p}^{2}$= 0.085). Participants in the Social group recalled more TBF items than participants in the Nonsocial group (*p* < 0.001). However, no difference was found between the two groups for TBR items (*p* = 0.421).

More importantly, a significant three-way interaction was found (*F*(1,80) = 4.071, *p =* 0.047, $\eta_{p}^{2}$ = 0.048). To further explore this interaction, we examined the *DF cue* × *TMS condition* interaction within each group. The results revealed that this two-way interaction was significant in the Nonsocial group (*F*(1,39) = 7.410, *p =* 0.010, $\eta_{p}^{2}$ = 0.160), but not in the Social group (*F*(1,41) = 0.017, *p =* 0.897, $\eta_{p}^{2}$= 0.000). Specifically, in the Nonsocial group, Active TMS improved recall for TBR items (*p* = 0.042), and showed a trend to reduce recall for TBF items (*p* = 0.060). Therefore, stimulating the rDLPFC increased the DF effect for nonsocial memories.

Table S1. Descriptive Statistics (Mean ± SD) for **Positive Valence** in Social and Nonsocial Groups.

|  | Social (*n* = 42) | | | | | |  | Nonsocial (*n* = 40) | | | | |
| --- | --- | --- | --- | --- | --- | --- | --- | --- | --- | --- | --- | --- |
|  | TBR | |  | TBF | | |  | TBR | |  | TBF | |
|  | Active | Control |  | Active | | Control |  | Active | Control |  | Active | Control |
| Sensitivity (d’) | 1.39 ± 0.94 | 1.53 ± 0.88 |  | 0.70 ± 0.71 | 0.86 ± 0.69 | |  | 1.66 ± 0.71 | 1.44 ± 0.82 |  | 0.56 ± 0.68 | 0.86 ± 0.67 |
| Recall accuracy | 0.34 ± 0.16 | 0.37 ± 0.19 |  | 0.15 ± 0.12 | 0.18 ± 0.14 | |  | 0.42 ± 0.18 | 0.35 ± 0.21 |  | 0.08 ± 0.08 | 0.11 ± 0.08 |
